# CSWS Versus SIADH as the Probable Causes of Hyponatremia in Children With Acute CNS Disorders

**Published:** 2013

**Authors:** Hadi SORKHI, Mohammad Reza SALEHI OMRAN, Rahim BARARI SAVADKOOHI, Farkhondeh BAGHDADI, Naeemeh NAKHJAVANI, Ali BIJANI

**Affiliations:** 1 Non-communicable Pediatric Disease Research Center, Department of Pediatric Nephrology, Amirkola Children Hospital, Babol Medical University, Babol, Iran; 2 Non-communicable Pediatric Disease Research Center, Department of Pediatric Infection disease, Amirkola Children Hospital, Babol Medical University, Babol, Iran; 3 Non-communicable Pediatric Disease Research Center, Department of Pediatric Intensive care unit, Amirkola Children Hospital, Babol Medical University, Babol, Iran

**Keywords:** Children, Acute CNS disorders, Cerebral salt wasting, Syndrome of inappropriate secretion of ADH

## Abstract

**Objective:**

There is a major problem about the incidence, diagnosis, and differentiation of cerebral salt wasting syndrome (CSWS) and syndrome of inappropriate secretion of antidiuretic hormone (SIADH) in patients with acute central nervous system (CNS) disorders. According to rare reports of these cases, this study was performed in children with acute CNS disorders for diagnosis of CSWS versus SIADH.

**Materials & Methods:**

This prospective study was done on children with acute CNS disorders. The definition of CSWS was hyponatremia (serum sodium ≤130 mEq/L), urine volume output ≥3 ml/kg/hr, urine specific gravity ≥1020 and urinary sodium concentration ≥100 mEq/L. Also, patients with hyponatremia (serum sodium ≤130 mEq/L), urine output < 3 ml/kg/hr, urine specific gravity ≥1020, and urinary sodium concentration >20 mEq/L were considered to have SIADH.

**Results:**

Out of 102 patients with acute CNS disorders, 62 (60.8%) children were male with mean age of 60.47±42.39 months. Among nine children with hyponatremia (serum sodium ≥130 mEq/L), 4 children had CSWS and 3 patients had SIADH. In 2 cases, the cause of hyponatremia was not determined. The mean day of hyponatremia after admission was 5.11±3.31 days. It was 5.25±2.75 and 5.66±7.23 days in children with CSWS and SIADH, respectively. Also, the urine sodium (mEq/L) was 190.5±73.3 and 58.7±43.8 in patients with CSWS and SIADH, respectively.

**Conclusion:**

According to the results of this study, the incidence of CSWS was more than SIADH in children with acute CNS disorders. So, more attention is needed to differentiate CSWS versus SIADH in order to their different management.

## Introduction

The concept of cerebral salt wasting syndrome (CSWS) was abandoned for a long time, despite it had been reported seven years before the syndrome of inappropriate secretion of antidiuretic hormone (SIADH) (1950 versus 1957) (1,2).

Hyponatremia is a common problem in central nervous system (CNS) disorders, and usually was attributed to SIADH (3-6). The main danger of hyponatremia is swelling of brain cells and increase of intracellular fluid (7-9). Therefore, early diagnosis and appropriate management, especially in children with acute neurologic disorders are of great importance. In SIADH, hyponatremia is caused by water retention due to inappropriate secretion of antidiuretic hormone (ADH) (10-15). But in CSWS, hyponatremia is associated with high urine output, high urine sodium concentration, and plasma volume depletion (16).

It is a major problem to distinguish CSWS from SIADH. Also, early diagnosis of them is very important, because SIADH is associated with volume retention, therefore, water restriction is the essential concept of their management (17,18). But CSWS is caused by the release of natriuretic peptides and hypovolemic hyponatremia that are associated with high urine volume and natriuresis. So, replacement of fluid and correction of hyponatremia are more important in CSWS (19). Although subarachnoid hemorrhage (SAH) is one the most common cause of CSWS (4), other acute CNS disorders, such as septic, viral, and herpetic meningitis have been reported as causes of CSWS (20-23)

There are rare reports about the incidence of hyponatremia in children with acute CNS disorders and also about SIADH versus CSWS. Moreover, SIADH may be more considered than CSWS and the diagnosis of CSWS is frequently missed by neurologists. So, this study was done on children with acute CNS disorders referred to Amirkola children hospital, Babol, Iran, with the aim of diagnosis of CSWS versus SIADH.

## Materials & Methods

In this prospective study, all children with acute CNS disorders who were admitted to Amirkola Children Hospital (from May 2010 to November 2011) were enrolled in the study.

Acute CNS disorders were as follows:

1. Status epileptics: convulsion more than 30 minutes;

2. Encephalopathy: the existence of at least two of the following symptoms that are presented by altered level of consciousness, cognition, personality, or seizures;

3. Encephalitis: encephalopathy and cerebral spinal fluid (CSF) pleocytosis;

4. Altered level of consciousness: increase or decrease of neuronal excitability that progress to coma;

5. Traumatic brain injury;

6. Aseptic meningitis: sign of meningismus and CSF leukocytosis without bacterial or fungal infection;

7. Septic meningitis: sign of meningismus and CSF leukocytosis with bacterial or fungal infection.

The definition of CSWS was hyponatremia (serum sodium ≤130 mEq/L), urine output ≥3 ml/kg/hr, urine specific gravity≥1020, and urinary sodium≥100 mEq/L (16). Also, patients with hyponatremia (serum sodium ≤130 mEq/L) according to every day serum sampling, urine output < 3 ml/kg/hr, urine specific gravity≥1020, and urinary sodium concentration > 20 mEq/lit were considered to have SIADH (11).

The exclusion criteria were as follows: all patients with history of endocrine, metabolic, renal or chronic neurologic disorders, and use of diuretic or manitol.

The data were analyzed by t-test using SPSS software. p<0.05 was considered statistically significant.

## Results

In this study, 102 patients with acute CNS disorders were included. Sixty-two (60.8%) patients were males and 40 (39.2%) were females.

Among these patients, 9 (8.8%, CI95%: 3.22-13.32%) children had hyponatremia (serum sodium <130 mEq/ lit). Four (3.92%, CI95%: 0.09-7.75%) had CSWS and 3 (2.9%, CI95%: 0.01- 6.28%) had SIADH. Also, 2 children had unknown cause of hyponatremia. The mean age of patients was 60.37±42.39 months (2-168 months). This was 93.40±40.31 months in children with CSWS and 96±43.26 months in patients with SIADH.

The most common causes of admission in children with acute CNS disorders were septic meningitis (31.4%) and traumatic brain injury (19.6%) (Table 1).

The mean serum level of sodium in all patients was 137±5.49 mEq/L (112-146 mEq/L). In patients with hyponatremia, the mean serum level of sodium was 124.7±5.9 mEq/lit. It was 124.1±8.2mEq/L in children with CSWS, and 124.8±4.5 mEq/L in patients with SIADH (P>0.05).

The mean day of hyponatremia after admission was 5.11±4.31 days. It was 5.25±2.75 and 5.66±7.23 days in children with CSWS and SIADH, respectively.

Among four patients with CSWS, 2 (50%) children had status epilepticus, and in three children with SIADH, there were status epilepticus, septic meningitis, and intracranial hemorrhage (Table 1).

The mean urine sodium (mEq/L) was 190.5±73.3 and 58.7±43.8 in patients with CSWS and SIADH, respectively (p<0.05). Also, the mean volume of urine (ml/kg/hr) in children with CSWS was more than SIADH (Table 2).

## Discussion

According to the findings of this study, 9 (55%) patients with acute CNS disorders had hyponatremia, and CSWS cases were more than SIADH. In Bussmann et al. study that was done on 195 children with acute CNS disorders for 5 years, 20 (10.3%) children had hyponatremia (serum sodium level ≤130 mEq/L); 9 (4.5%) children had CSWS; and 7 (3.5%) had SIADH. Therefore, the rate of CSWS was more than SIADH (24). Jimenez reported 14 (1.13%) children with CSWS in 1229 patients (less than 15 years old) after neurosurgery and after admission to pediatric intensive care unit (PICU) (16).

In other study on 282 children (291 neurosurgery patients due to brain tumors), CSWS was detected in 15 (5%) cases, and 9 (3%) patients had SIADH (25). Although, in Agha et al.’s study that was done on 316 patients with subarachnoid hemorrhage (SAH), 179 (56.6%) patients had hyponatremia (serum sodium level ≤135 mEq/L). The causes of hyponatremia were SIADH and CSWS in 39 (62.9%) and 4 (6.5%) patients, respectively (26). However, there are many studies that were recommended CSWS does not really exist and these patients may be in SIADH category (11,27,28). For example, in a study performed on 40 patients with hyponatremia and suspected SIADH or CSWS (in ICU), there were not any cases with diagnosis of CSWS (11). Also, Singh et al. reported that CSWS is very rare and less common than SIADH (29). But there are some studies that reported CSWS may be more common than SIADH in patients with SAH and intracranial infection (encephalitis, meningitis (20-23,30).

The causes of hyponatremia in CNS disorders and especially after neurosurgery are different and may be related to over administration of hypotonic fluid, use of diuretic, SIADH, CSWS, hypothyroidism, as well as renal, liver, or adrenal insufficiently (19).

It is very important to differentiate the causes of hyponatremia (especially SIADH versus CSWS), because there are different management for them (19,31,32).

Both disorders have high urine osmolality and increase of specific gravity, but in SIADH, it is due to inappropriate secretion of antidiuretic hormone (ADH), and in CSWS is associated with volume contraction. Also, urinary sodium loss is high in both disorders, but it is higher in CSWS (32).

The most important finding for differentiation of CSWS from SIADH is decrease in blood volume (hypotension, decreased skin turgor, and increased hematocrite) with high urine sodium concentration. However, patients with SIADH may have normal or mild increase in blood volume (13,27).

The pathogenesis of CSWS is not clear. Some important factors are: arterial natriuretic peptide (ANP), brain natriuretic factor (BNP), C-type natriuretic factor (CNP) and dendroaspis natriuretic peptide (DNP), but the role of BNP is more important (33-37). Also, in spite of increase in natriuretic peptides, other mechanisms may be important for pathogenesis of CSWS, such as abnormality of sympathetic nervous system and increase in natriuresis (19).

The different incidence of CSWS versus SIADH in patient with acute neurologic disorder may be due to different criteria for differentiation of CSWS and SIADH. For example, in some studies, the definition of hyponatremia was “serum sodium≤of 135 mEq/L” and in some others, it was serum sodium≤130 mEq/L. In one study, the definition of CSWS was negative blood volume balance more than 20%, or increase of hematocrite without administration of transfusion, and in another study, urinary sodium concentration more than 120 mEq/L, urinary osmolality more than 300 mOsm/kg H2O, urine volume more than 2-3 ml/kg/hr were criteria for CSWS. In one study, fraction excretion of uric acid more than 10% with natriuresis and decrease of blood volume were used for definition of CSWS and in another study, increase in urinary sodium and chloride excretion were used for definition of CSWS. Also, central venous pressure (CVP) was used for determination of blood volume (16,20,24,38,39).

In spite of different definition of CSWS, the existence of CSWS (even more than SIADH) in our study indicated the importance of early diagnosis and making a good plan for its management.

Among 9 Children with hyponatremia, 3 (30%) patients had status epilepticus, that 2 cases had CSWS and one had SIADH. In Jiménez et al.’s study, brain tumor was the most common causes of hyponatremia (16). SAH was the most common cause of hyponatremia in Bussmann et al.’s report and the majority of their patients with CSWS had neurosurgery operation for brain tumor (24). Our hospital is a referral children hospital and the majority of patients have non-surgical problem. Therefore, the difference in incidence and cause of hyponatremia may be due to our different referral patients.

In our study, 5 patients with hyponatremia were female and in 2 patients with hyponatremia the cause was unknown. Among 16 patients with hyponatremia and diagnosis of CSWS or SIADH, 8 patients were female (24). In another study, 9 children with hyponatremia and acute CNS disorders were males and 6 patients were female (16). Therefore, it seems that the risk of hyponatremia is not different between two sexes.

**In conclusion** **, **in summary, there is a risk of hyponatremia in different disorders of acute CNS diseases. Although there were small numbers of both CSWS and SIADH patients, but the risk of CSWS is more than SIADH in children with acute neurologic disorders. So, according to different managements of these disorders, more attention is needed for differentiation of CSWS versus SIADH.

**Table 1:** Frequency of Primary Acute CNS Disorders in Children Who Referred to Amirkola Children Hospital

**Primary disease**	**Frequency**
Status epilepticus	9 (8.8%)
Encephalitis	4(3.9%)
Encephalopathy	17(16.7%)
Altered level of Contionesness	6 (5.9%)
Traumatic brain injury	20(19.6%)
Aseptic meningitis	14(13.7%)
Septic meningitis	32(31.4%)
Total	102

**Table 2:** Characteristics of Patients With Hyponatremia in Children with Acute CNS Disorders Referred to Amirkola Children Hospital According to Diagnosis

**Age** **(Months)**	**Sex**	**Primary disorders**	**Serum Na level**	**Days of hyponatremia**	**Final diagnosis of hyponatremia**
2.5	Male	Head Trauma	122	1	Unknown
60	Female	Septic meningitis	128	14	ASIDH
62	Male	Head Trauma	129	7	CSWS
144	Female	Status epilepticus	123.2	1	SIADH
84	Female	Intracranial hemorrhage	125.5	2	SIADH
120	Male	Encephalopathy	129.7	7	Unknown
60	Male	Status epilepticus	129	4	CSWS
144	Female	Status epilepticus	127	2	CSWS
108	Female	Intracranial hemorrhage	112	8	CSWS

**Table 3:** Laboratory Characteristics of Children with Acute CNS Disorders with Hyponatremia (CSWS and SIADH) Referred to Amirkola Children Hospital

**Characteristics**	**CSWS Mean±SD**	**SIDAH Mean±SD**
Serum Sodium (mEq/lit)	124.1± 8.2	124.8± 4.5
Urine Specify gravity	1025/75 ± 4.19	1024.66± 7.57
Serum Osmolallity (mOsmol/kg H o) 2	247.15±15.62	246± 4.21
Urine Volume (ml/kg/hr)	4.24±1.44	2.77± 0.32
Urine Sodium (mEq/lit)	190.5±73.3	58.7± 43.8*
Serum Uric Acid (mg/dl)	1.25±0.07	4.43±2.45
Serum Creatinine (mg/dl)	0.55 ± 0.12	0.64±0.05
Serum Potassium (mEq/L)	3.86±0.94	3.83±0.97

*p<0.05
